# Blood Morphological and Biochemical Indicator Characteristics in Men Performing Different Physical Activities in the Cold—A Preliminary Report

**DOI:** 10.3390/life14040474

**Published:** 2024-04-04

**Authors:** Aneta Teległów, Wacław Mirek, Bartłomiej Ptaszek, Marcin Maciejczyk, Dorota Godawska, Jakub Marchewka

**Affiliations:** 1 Department of Health Promotion, Institute of Basic Sciences, University of Physical Education, 31-571 Krakow, Poland; 2 Institute of Sports Science, University of Physical Education, 31-571 Krakow, Poland; waclaw.mirek@awf.krakow.pl; 3 Institute of Applied Sciences, University of Physical Education, 31-571 Krakow, Poland; 4 Department of Physiology and Biochemistry, University of Physical Education, 31-571 Krakow, Poland; marcin.maciejczyk@awf.krakow.pl; 5 Civilization Diseases Treatment Centre, Specialist Clinics Complex, 31-559 Krakow, Poland; d.godawska@gmail.com; 6 Institute of Clinical Rehabilitation, University of Physical Education, 31-571 Krakow, Poland; jakub.marchewka@awf.krakow.pl

**Keywords:** winter swimming, outdoor amateur running, blood morphological and biochemical indicators

## Abstract

This descriptive study determined whether winter swimming (WS) and outdoor amateur running (RUN) affect blood morphological and biochemical indicators in men during midseason winter swimming from November to April. There were three groups of participants, with 10 male amateurs each: RUN + WS, WS, and control. The research was performed in the middle of the winter swimming season of 2020/2021. This time period was chosen in consideration of the respondents’ adaptation to winter conditions. The study involved only 10 male amateurs in each study group owing to COVID-19 pandemic restrictions, which confined people to their homes. In the RUN + WS group compared with the WS group, significant decreases in the mean corpuscular hemoglobin concentration (within standard limits) (*p* = 0.04) and platelet distribution width (*p* = 0.006) were observed, with a significant increase in the red blood cell distribution width (*p* = 0.008) (within standard limits). The renal function, as expressed by the estimated glomerular filtration rate, was higher in the RUN + WS group (*p* = 0.02) (within standard limits) compared with the WS group, and the uric acid concentration was reduced (*p* = 0.01). In the RUN + WS group compared with the control group, significant decreases in the leukocyte count (*p* = 0.02) (within standard limits), monocyte count (*p* = 0.04) (within standard limits), and platelet distribution width (*p* = 0.005) were reported. The remaining indicators presented a *p*-value > 0.05. The two investigated forms of physical activity had no negative effect on blood morphological or biochemical indicators in male amateurs during the winter swimming midseason.

## 1. Introduction

The combination of physical activity (i.e., running) and winter swimming improves physical conditions and the capacity of numerous body systems, as well as mental conditions [1,2]. Lateef [1] indicates that taking a postexercise plunge into an ice bath at 12–15 °C appears to be a common practice among many elite athletes. This is believed to reduce muscle pain and soreness after training sessions and competitions.

Running for as little as 5–10 min a day at a speed not exceeding 10 km/h is associated with significant reductions in the risks of death and cardiovascular disease [3]. Physical activity of less than 1 h per week provides significant benefits to healthy people with sedentary lifestyles [4]. Winter swimmers maintain that contact with icy water is good for their health [5]. These findings suggest that regular exposure to the cold can be effective in the treatment of chronic autoimmune inflammation, reduce hypercholesterolemia by brown adipose tissue activation, and have a positive effect on stress regulation [6].

Physical activity combined with winter swimming reduces the risks of infections and colds. In Poland, according to the MultiSport Index 2022 report [7], amateur runners are the largest group among those involved in amateur sports [8], and winter swimming became a ‘national sport’ during the COVID-19 pandemic.

Poles eagerly practice winter swimming, treating it as a form of recreation that causes positive changes in both the body and mind. Winter swimming involves regular immersion or short-term bathing in natural bodies of cold water, such as lagoons, rivers, ponds, waterfalls, seas, lakes, or backyard water barrels.

Winter swimming exerts highly beneficial effects on the rapid regeneration of the human body. Many runners find that a few minutes of bathing in cold water definitely reduces pain after long runs [7]. An ice bath causes the constriction of blood vessels, which has been suggested as a mechanism that helps to flush waste products, such as lactic acid, out of affected tissues. In the cold temperatures, a reduction in metabolism is observed, and this can slow down physiological processes. The cold temperatures reduce swelling and tissue breakdown, and ice water immersion is also said to be able to shift lactic acid.

Winter swimming combines the effect of cold, water, physical effort, and endorphins, thus improving health, thermoregulation, blood circulation, blood supply to the skin, well-being, and vitality [2,9,10]. Previously, Teległów et al. [11] evaluated the morphological and biochemical blood properties of a multiple Guinness World Record holder, Valerjan Romanovski, who was exposed to an extremely cold environment, with temperatures ranging from +2 °C to −37 °C, for 50 days in Rovaniemi (a city in northern Finland). They concluded that the subject’s long-term exposure to extreme stress due to cold temperatures did not have a noticeably negative impact on daily functioning. The stimulating factors not only included the extreme cold but also the physical activity and other methods of keeping warm [11].

The body’s response to low temperature involves hormonal changes in the circulatory, nervous, and muscular systems [2]. According to Teległów et al. [12], regular immersion in cold water (e.g., winter swimming) increases red blood cell deformability in a constricted blood vessel system after a whole season of winter swimming, with no accompanying changes in erythrocyte aggregation (aggregation index, total extent of aggregation, half time kinetics of aggregation, blood plasma viscosity, and fibrinogen concentration). The increased erythrocyte elasticity in winter swimmers is a form of protection that facilitates the flow of blood cells in constricted blood vessel systems. Erythrocytes, during their multiple passages throughout the circulatory system (they cover a distance of approximately 400 km per day, or 190,000 km over their lifetime), must efficiently perform their primary function of transporting respiratory gases, i.e., oxygen and carbon dioxide [13]. In order to accomplish this, in the smallest vessels, which have diameters of 1–3 µm, forming the capillary network, red blood cells must exhibit sufficient elasticity, called deformability in rheology. The specific structure of erythrocytes provides them with approximately 40% excess surface area in relation to their volume, allowing them to repeatedly deform and return to their initial shape [14]. The main determinants of deformability are the cell shape (the ratio of the surface area to volume), intrinsic viscosity, and cell membrane elasticity.

Among winter swimmers, red blood cell deformability plays a significant role in blood flow not only during the winter swimming season but also after its completion [15]. In spring and summer, after incorporating other forms of physical activity, they were found to maintain the deformability of their red blood cells without changes in their aggregation. The studied group of winter swimmers were well trained and, during the offseason, actively participated in other sports, such as jogging, cycling, canoeing, trekking, and swimming, which significantly affected the maintenance of blood rheological properties. This increased elasticity can help improve blood flow and reduce the risk of circulation problems, contributing to overall cardiovascular health. Wesołowski et al. [16] demonstrated that repeated winter swimming resulted in the effective removal of lipid peroxidation products. Swimming in ice-cold water has also been shown to have a positive impact on mental health status [17] and to exert a potential antidepressive influence [18]. Checinska-Maciejewska et al. [19] suggest that the favorable effect of cold swimming on cardiovascular risk factors may be sex-dependent.

The study aimed to compare the effect of two forms of physical activity (winter swimming and outdoor amateur running) on blood morphological and biochemical indicators in men in the middle of the winter swimming season. We hypothesized that the physiological response to cold might be different after running than after swimming in the cold.

## 2. Material and Methods

### 2.1. Participants

The research was performed in the middle of the winter swimming season of 2020/2021. The time point was chosen in consideration of the respondents’ adaptation to winter conditions. This descriptive study involved only 10 male amateurs in each study group, as owing to the COVID-19 pandemic restrictions, people were confined to their homes. The 2020/2021 season brought a significant increase in the popularity of winter swimming in Poland; this is strongly linked to the COVID-19 pandemic restrictions. The inability to perform some previously practiced forms of recreation led people to consider new ways of spending their leisure time; some combined running with winter swimming.

The main inclusion criterion was the subjects’ activity level. To determine the outdoor running intensity, the participants used heart rate monitors manufactured by Polar (Polar M400, POLAR, Kempele, Finland) and Garmin (Forerunner 225, Garmin International, Kansas City, MO, USA). Maximum heart rate was calculated with the formula HRmax = 220 − age. Prior to the start of the study, the recruitment of willing participants was carried out by presenting the basic selection criteria for the study groups. Additionally, the exclusion criteria were the following: diabetes mellitus, use of beta-blockers or antidepressants, smoking or chewing tobacco products, and consuming more than 4 cups of coffee or more than 2 alcoholic beverages a day. The respondents were residents of Krakow (Poland), performing both physical and mental work. They were involved in the research program after obtaining a physician’s consent and after a physiotherapist’s consultation. Before enrolment, each volunteer read the patient information leaflet and, if any doubts appeared, they could ask questions.

The first group, who combined outdoor running (jogging; 1 h 3 times per week) and regular winter swimming (3 min 3 times per week) (RUN + WS group), included male amateurs (*n* = 10) from the Krakow Society of Winter Swimmers ‘Kaloryfer’ in Krakow (Poland), practicing at the Bagry Lagoon. The respondents ran 45’45’’ (standard deviation: 3’11’’) on average for 10 km. The jogging was performed at an intensity of up to 85% maximum heart rate; in accordance with the Polish school, this was the so-called intensity range 2, which corresponds to intensity zone 3 proposed by Seiler and Tønnessen [20].

The second study group consisted of male amateurs (*n* = 10) from the same association who had regularly practiced winter swimming for several years at the Bagry Lagoon (3 min 3 times per week) (WS group).

The control group participants (*n* = 10) had never performed winter swimming or running; they led a sedentary lifestyle.

Poland is located in Europe in a temperate climate zone with mixed continental and oceanic influences. The mean air and water temperatures during the 2020/2021 winter swimming season are presented in Table 1.

The study participants’ age, body height, and body mass are shown in Table 2. Body mass was measured with Model Tanita BC-418 MA 3 scales (TANITA Corporaton, Sportlife Tokyo, Japan).

### 2.2. Blood Sample Collection

Fasting blood samples were taken from the participants of all groups once, in the middle of the winter swimming season 2020/2021 (January) at week 13 from the start of the winter swimming season, in the morning, in an amount of 10 mL into Vacuette EDTA K2 tubes and into tubes with clotting activator. The time point of the middle of the season was chosen because of the subjects’ adaptation to winter conditions. Blood was collected in the RUN + WS and WS groups after the same number of weeks of training. The blood samples were taken only once because of the COVID-19 pandemic restrictions.

The blood samples were collected by a qualified nurse at the Blood Physiology Laboratory of the University of Physical Education in Krakow. The material analyses were performed in the Department of Clinical Biochemistry of the Krakow Branch of Maria Skłodowska-Curie National Research Institute of Oncology. The study was approved by the Ethics Committee of the Regional Medical Chamber in Krakow, Poland (approval No.: 194/KBL/OIL/2019).

### 2.3. Morphological and Biochemical Assays

Complete blood count was performed with an ADVIA 2120i analyzer (Siemens, Healthcare Diagnostics, Deerfield, IL, USA) and involved red blood cell (RBC) count [×10^12^/L], hemoglobin (Hb) concentration [g/dL], hematocrit (HCT) [%], mean corpuscular volume (MCV) [fL], mean corpuscular hemoglobin (MCH) [pg], mean corpuscular hemoglobin concentration (MCHC) [g/dL], corpuscular hemoglobin concentration mean (CHCM) [g/dL], red blood cell distribution width (RDW) [%], hemoglobin distribution width (HDW) [g/dL], white blood cell (WBC) count [×10^9^/L], neutrocyte count [×10^9^/L], eosinocyte count [×10^9^/L], basophil count [×10^9^/L], lymphocyte count [×10^9^/L], monocyte count [×10^9^/L], platelet (PLT) count [×10^9^/L], mean platelet volume (MPV) [fL], plateletcrit (PCT) [%], platelet distribution width (PDW) [%].

To measure electrolytes, a Cobas device (Roche Diagnostics GmbH, Mannheim, Germany) was used with an ion-selective electrode module to quantify potassium, sodium, and chloride ions. The sodium and potassium electrodes are based on natural carriers, and the chloride electrode is based on an ion exchanger. The following electrolytes were analyzed: Na^+^ [mmol/L] (concentration of sodium ions), K^+^ [mmol/L] (concentration of potassium ions), and Cl^−^ [mmol/L] (concentration of chloride ions).

The renal and liver profiles were determined with the colorimetric method by using reagent kits and a Cobas c 311 analyzer (Roche Diagnostics). For each sample, the device automatically calculates the analytical activity of the given substance. In the renal profile, urea [mmol/L], creatinine [mmol/L], estimated glomerular filtration rate (eGFR) [mL/min/1.73 m^2^], and uric acid [μmol/L] were measured. In the liver profile, total bilirubin [μmol/L], alkaline phosphatase (ALP) [U/L], aspartate transaminase (AST) [U/L], alanine transaminase (ALT) [U/L], and gamma-glutamyltransferase (GGT) [U/L] were evaluated.

A Dade Behring device in the BN ProSpec system was applied to assess the diagnostic indicators for determining immunoglobulins. The immunonephelometric method served to investigate immunoglobulins (IgG, IgA, IgM) in human serum with reagents designed for in vitro diagnostics. C-reactive protein (CRP; an acute-phase protein) concentration was evaluated with the immunonephelometric method, by using reagent kits and a BN ProSpec nephelometer (Siemens, Healthcare Diagnostics, Deerfield, IL, USA). Protein electrophoresis was performed in a Cobas c 311/511 analyzer in Roche/Hitachi systems.

The concentrations of albumin and the other fractions (alpha-1-globulin, alpha-2-globulin, beta-1-globulin, beta-2-globulin, gamma-globulin) were calculated on the basis of the total protein concentration and the percentage of electrophoretic fraction, determined in the analysis of electropherograms, obtained after separation of serum proteins by capillary electrophoresis (Minicap, Sebia, Italia Srl).

### 2.4. Statistical Analysis

The results are presented as the median and standard deviation. Owing to a small sample size, in order to assess the significance of differences among groups, nonparametric analysis of variance (the Kruskal–Wallis test) was used. In the case of significant differences, a post hoc analysis (i.e., a comparison between 2 particular groups) was carried out with the Mann–Whitney test. The statistical analysis of the results was performed using Statistica 12.0 software (StatSoft Inc., Tulsa, OK, USA). Differences, for all analyzed indicators, were considered statistically significant at the level of *p* < 0.05.

## 3. Results

The medians of the obtained results for the blood morphological and biochemical indicators are presented in Table 3 and Table 4, respectively.

### 3.1. Morphological Indicators: RUN + WS vs. WS

In the two analyzed groups, no statistically significant changes were found for the mean values of the blood morphological indicators (Table 3). The only observed differences between the RUN + WS group and the WS group were a significant decrease in the MCHC (*p* = 0.04) and PDW (*p* = 0.006) and a significant increase in the RDW (*p* = 0.008).

### 3.2. Morphological Indicators: RUN + WS vs. Control

In the two analyzed groups, no statistically significant changes were found for the mean values of blood morphological indicators (Table 3). The only statistically significant differences between the RUN + WS group and the control group were lower (within standard limits) WBC (*p* = 0.02), monocyte (*p* = 0.04), and PDW (*p* = 0.005) values (Table 3).

### 3.3. Morphological Indicators: WS vs. Control

In the two analyzed groups, no statistically significant changes were found for the mean values of blood morphological indicators (Table 3). The only statistically significant differences between the WS group and the control group were higher (within standard limits) MCHC (*p* = 0.03) and RDW (*p* = 0.003) values, as well as a lower MCV (*p* = 0.008).

### 3.4. Biochemical Indicators: RUN + WS vs. WS

In the two analyzed groups, no statistically significant changes were found for the mean values of blood biochemical indicators (Table 4). The only statistically significant differences between the RUN + WS group and the WS group (within standard limits) were increased eGFR (*p* = 0.02) and decreased uric acid concentration (*p* = 0.01) values.

### 3.5. Biochemical Indicators: RUN + WS vs. Control

In the two analyzed groups, no statistically significant changes were found for the mean values of blood biochemical indicators (Table 4). The only statistically significant differences between the RUN + WS group and the control group (within standard limits) were higher Na^+^ (*p* = 0.005), Cl^−^ (*p* = 0.02), and ALP (*p* = 0.04) values.

### 3.6. Biochemical Indicators: WS vs. Control

In the two analyzed groups, no statistically significant changes were found for the mean values of blood biochemical indicators (Table 4). The only statistically significant differences between the WS group and the control group (within standard limits) were higher ALP (*p* = 0.02) and beta-1-globulin (*p* = 0.02) values.

## 4. Discussion

The aim of the study was to compare blood morphological and biochemical indices in men exercising in the cold but undertaking different forms of physical activity. We expected that there would be significant differences in the studied parameters between the groups; however, only significant changes within standard limits were observed. Physical activity in the cold, regardless of the form, also did not cause any negative effects, which indicates good thermoregulation of the body, increased resistance to hypothermia, and improved cold tolerance. Cold exposure provided positive effects in the WS and the WS + RUN groups.

One important factor sustaining physical and mental well-being [21] and enabling the process of successful aging is an adequate level of physical activity [22,23]. Regular winter swimming and amateur running have an impact on efficient blood flow through the arteries, veins, and capillaries. Blood morphological and biochemical indicators in amateur runners of different ages can well characterize the actual extent and direction of effort changes in conjunction with winter swimming, as evidenced in the presented study (all blood test results remained within the accepted reference ranges for the human population). Winter swimming combines the influence of cold, water, physical activity, and climate factors resulting in a strong stimulus, although one should bear in mind the numerous contraindications (e.g., circulatory and respiratory insufficiencies). According to Pournot et al. [24], repeated sessions of whole body cryotherapy (3 min at −110 °C) can improve recovery, as they decrease the acute-phase inflammatory response after running and play a beneficial role in organ protection after muscle damage. The results demonstrated that whole body cryotherapy was effective in inflammatory process reduction, which may be explained by vasoconstriction at the muscular level, as well as decreased pro-inflammatory cytokine activity and increased anti-inflammatory cytokine activity [24].

It should be emphasized that regular moderate physical effort stimulates immune mechanisms in combination with winter swimming [25,26]. Therefore, homeostatic alterations in response to aggressive situations, such as intense physical exercise, seem to be attenuated by the application of previous moderate physical training. The cells of the immune system seem to present adaptative tolerance mechanisms that allow for improvement in their function in response to regular physical exercise of moderate intensity [25]. A regular exercise practice promotes improvement in quality of life and can influence the immune response, reducing the risk of developing systemic inflammatory processes and stimulating cellular immunity [27]. As implied by Lombardi et al. [28], winter swimmers have significantly higher leukocyte and PLT counts and subpopulations of neutrophils, lymphocytes, and monocytes. The authors revealed that these increases in the measurements reflected direct hematopoiesis induction at the bone marrow level. This resulted from sympathetic activity and the activation of numerous hormonal mediators regulating energy metabolism and cold adaptation (among them, leptin). Swimming in cold water decreased plasma volume (on the basis of hemoglobin and hematocrit variations) [28]. In the present study, no statistically significant differences were found in the red blood cell, white blood cell, or platelet systems in the investigated groups (i.e., RUN + WS, WS, and control) for RBC, Hb, HCT, MCH, neutrocyte count, eosinocyte count, basophil count, lymphocyte count, PLT, MPV, or PCT.

The differences in the reports result from variable exposure of the participants to low temperatures and a dissimilar response of the sympathetic nervous system activation. Most likely, the minor differences in blood morphological indicators were due to the body adaptation to both winter swimming in the WS group and running combined with winter swimming in the RUN + WS group amateurs. The slight differences in blood morphological indicators within standard limits are most probably caused by variations in the circulating blood volume. For example, Janský et al. [29] observed minimal changes in the number of immune cells after a single immersion in cold water and reported no statistically significant changes in leukocytes after a 6-week series of cold water baths. Blood morphological indicators in the examined individuals provide a good description of the actual extent and direction of changes and allow for the diagnose of transient adaptive effects. In the RUN + WS group compared with the WS group and control group, a decrease in PDW was observed; both results, however, exceeded the laboratory norm, which, in the absence of changes in the PLT and MPV values, implies that this condition was not clinically significant. Similar results to those obtained in the present study for the WS group compared with the control group for MCHC were reported by D’Alesandro et al. [30], who demonstrated increased MCHC in a group of men repeatedly exposed to a temperature of 4 °C for 30 min. In turn, Banffi et al. [31] and Wcisło et al. [32] observed a reduced MCHC; Lombardi et al. [28] noted no variations in the hematological indicators of the MCH, MCV, or MCHC. However, in the present study, the MCHC values were lower in the RUN + WS group than in the WS group; in turn, the MCV was significantly lower in the WS group than in the control group.

A previous study by Teległów et al. [15] confirmed that winter swimming did not alter the biochemical parameters constituting the renal and hepatic profiles, which was most likely due to long-term adaptation to these conditions. It is important to emphasize that the liver is one of the main organs that warm the blood and, therefore, the entire body, which is critical in low temperatures. Significant decreases in Na^+^ and Cl^−^ concentrations were observed in the study, and the most probable explanation for these phenomena is the loss of these electrolytes in the urine as a result of increased diuresis after a cold bath, which, in turn, is due to central blood redistribution caused by peripheral vasoconstriction and increased blood pressure. No statistically significant differences were reported for CK. It is widely recognized that IgA, owing to its dominance in the immune system, is the first line of defense against harmful environmental agents. This study revealed a slight increase in IgA after regular winter swimming sessions and a minor decrease in gamma-globulins, including IgG, and alpha-2-globulin, which are associated with immunity acquired by winter swimmers. Wesołowski et al. [16] confirmed that regular winter swimming effectively removed lipid peroxidation products.

In the present study, no statistically significant differences were shown for the RUN + WS, WS, and control groups with reference to the following blood biochemical indicators: K^+^, urea, creatinine, total bilirubin, AST, ALT, GGT, LDH, CK, total protein, albumin, alpha-1-globulin, alpha-2-globulin, beta-2-globulin, gamma-globulins, A/G, IgA, IgG, IgM, and CRP. Similar results were achieved by Teległów et al. [15], Siems et al. [26], and Janský et al. [29], who reported no significant changes after repeated cold water immersions for interleukin 6, IgG, IgM, IgA, or CRP. The conclusion was that the immune system was slightly activated by the stress-inducing stimulus of repeated cold water immersions, which raised the metabolic rate because of shivering and elevated blood catecholamine concentrations.

In the RUN + WS group compared with the WS group, there were statistically significant differences within standard limits for eGFR and uric acid concentration. Renal function, expressed by eGFR, was higher in the RUN + WS group and the uric acid concentration was lower. This implies that the participants who combined winter swimming and amateur running experienced fluid shifts between the intravascular and interstitial spaces, fluid loss with sweat, and changes in some hormone concentrations, e.g., increases in adrenaline, glucagon, cortisol, or adrenocorticotropic hormone. According to Siems et al. [26], changes in uric acid concentrations during ice swimming suggest that intense, voluntary, and short-term exposure to cold during winter swimming causes oxidative stress.

In the RUN + WS group compared with the control group, the following differences within standard limits were observed: increases in Na^+^ and Cl^−^, as well as a decrease in ALP. Fluctuations (within standard limits) in the concentrations of Na^+^ and Cl^−^ ions are dependent on the type and amount of fluids ingested, and ALP reduction (within standard limits) indicates nutritional deficiencies.

The comparison of the WS group and the control group revealed a decrease in ALP (within standard limits) and an increase in beta-1-globulin (exceeding standard limits), implying an impact of winter swimming on the immune system.

The comparison of the effect of two forms of physical activity (winter swimming and amateur running) in men during the middle of the winter swimming season on blood morphological and biochemical indicators reveals that all blood test results remained within the accepted reference ranges for the human population.

A limitation of the study is the sample size due to the small number of participants who both swim and run in the cold and who practice only winter swimming. These activities are not very popular. The study evaluated morphological and biochemical blood indicators at a single point of measurement during the middle of the winter season. This allowed for the assessment of the health status of the participants after regular activity in the cold; however, it did not allow for evaluations of the effect size of this activity (pre vs. post).

## 5. Conclusions

The investigated forms of physical activity (outdoor amateur running and winter swimming) had no negative effect on blood morphological or biochemical indicators in male amateurs during the middle of the winter swimming season. The indicators studied were within physiological norms, and the type of physical activity did not have a more favorable effect on the analyzed parameters. Combining high-intensity running (even 85% HRmax) with winter swimming can serve as a suggested supplemental physical activity. As studies confirm, it has a positive health aspect and is a future direction for research concerning both sexes.

## Figures and Tables

**Table 1:** Mean air and water temperatures in Poland during the 2020/2021 winter swimming season.

Month	Mean Air Temperature in Poland	Mean Water Temperature in Poland
November	5.6 °C	7 °C
December	1.9 °C	4 °C
January	−0.5 °C	4 °C
February	0.1 °C	4 °C
March	5.1 °C	4 °C
April	6.0 °C	4 °C

**Table 2:** Characteristics of the investigated groups.

Parameter	RUN + WS Group (*n* = 10) M (Min–Max) ± SD	WS Group (*n* = 10) M (Min–Max) ± SD	Control Group (*n* = 10) M (Min–Max) ± SD	*p* (ANOVA on Ranks)	Post Hoc Analysis		
					RUN + WS /WS	WS/CON	RUN + WS /CON
Age [years]	38.5 (27–46) ± 3.79	40.0 (34–44) ± 1.57	41.0 (35–45) ± 0.34	0.07	n.s.		
Body height [cm]	177.5 (176–178) ± 1.04	177.5 (173–183) ± 2.26	176.5 (175–186) ± 2.27	0.97	n.s.		
Body mass [kg]	71.5 (70–74) ± 6.44	77.5 (74–96) ± 5.84	76.7 (68–80) ± 2.32	0.06	n.s.		

M—median; RUN—running; WS—winter swimming; CON—control; n.s.—no significant difference.

**Table 3:** Median blood morphological indicators in the investigated groups.

Parameter	RUN + WS Group (*n* = 10) M (Min–Max) ± SD	WS Group (*n* = 10) M (Min–Max) ± SD	Control Group (*n* = 10) M (Min–Max) ± SD	*p* (ANOVA on Ranks)	Post Hoc Analysis		
					RUN + WS/ WS	WS/CON	RUN + WS /CON
RBC [×10^12^/L]	5.05 (4.58–5.25) ± 0.14	5.11 (4.9–5.22) ± 0.13	5.23 (4.88–5.32) ± 0.11	0.58	n.s.		
Hb [g/dL]	15.15 (14–15.4) ± 0.22	14.75 (14.4–15.8) ± 0.42	15.50 (14.9–16) ± 0.33	0.31	n.s.		
HCT [%]	45.60 (42.9–46.2) ± 0.68	44.30 (42.4–47) ± 1.16	46.75 (45.7–49.2) ± 0.98	0.12	n.s.		
MCV [fL]	90.85 (87.7–94.3) ± 1.29	87.25 (85.3–89.1) ± 1.2	90.40 (89.4–93.2) ± 0.76	0.03 *	0.09	0.008 *	0.67
MCH [pg]	30.50 (29.2–31.1) ± 0.5	29.35 (28.7–29.7) ± 0.35	29.95 (29.8–30.4) ± 0.14	0.06	n.s.		
MCHC [g/dL]	33.0 (32.9–33.3) ± 0.08	33.70 (33.1–34.1) ± 0.21	32.95 (32.6–33.4) ± 0.16	0.04 *	0.04 *	0.03 *	0.7
RDW [%]	15.20 (13.3–15.6) ± 0.43	12.85 (12.6–13.3) ± 0.15	12.80 (12.4–13) ± 0.15	0.003 *	0.008 *	0.003 *	0.38
WBC [×10^9^/L]	4.23 (3.99–5.99) ± 0.49	5.09 (3.77–6.08) ± 0.49	6.35 (5.98–6.89) ± 0.39	0.04 *	0.79	0.053	0.02 *
Neutrocytes [×10^9^/L]	1.85 (1.6–3.03) ± 0.33	2.69 (1.82–2.75) ± 0.3	3.07 (2.56–3.6) ± 0.32	0.09	n.s.		
Eosinocytes [×10^9^/L]	0.12 (0.07–0.16) ± 0.03	0.16 (0.12–0.21) ± 0.02	0.16 (0.03–0.06) ± 0.05	0.63	n.s.		
Basophils [×10^9^/L]	0.04 (0.03–0.05) ± 0.01	0.03 (0.03–0.04) ± 0.003	0.04 (0.03–0.06) ± 0.01	0.2	n.s.		
Lymphocytes [×10^9^/L]	1.92 (1.56–2.43) ± 0.15	1.61 (1.37–2.69) ± 0.22	2.26 (1.73–2.46) ± 0.21	0.25	n.s.		
Monocytes [×10^9^/L]	0.41 (0.28–0.47) ± 0.03	0.36 (0.3–0.49) ± 0.03	0.50 (0.43–0.63) ± 0.05	0.04 *	0.96	0.02 *	0.04 *
PLT [×10^9^/L]	204.0 (193–263) ± 15.97	252.5 (200–262) ± 16.6	218.5 (181–243) ± 18.96	0.4	n.s.		
MPV [fL]	8.40 (7.2–9) ± 0.67	9.16 (8.6–10.2) ± 0.32	9.30 (8.7–9.9) ± 0.01	0.21	n.s.		
PCT [%]	0.20 (0.18–0.25) ± 0.09	0.21 (0.19–0.25) ± 0.01	0.19 (0.18–0.21) ± 0.01	0.59	n.s.		
PDW [%]	19.10 (17.2–48.9) ± 5.45	57.90 (48.6–60.6) ± 2.22	55.05 (52–56) ± 1.98	0.004 *	0.006 *	0.38	0.005 *

M—median; RUN—running; WS—winter swimming; CON—control; RBC—red blood cell count; Hb—hemoglobin concentration; HCT—hematocrit; MCV—mean corpuscular volume; MCH—mean corpuscular hemoglobin; MCHC—mean corpuscular hemoglobin concentration; RDW—red blood cell distribution width; WBC—white blood cell count; PLT—platelet count; MPV—mean platelet volume; PCT—plateletcrit; PDW—platelet distribution width; n.s.—no significant difference. * Significant difference (*p* < 0.05).

**Table 4:** Median blood biochemical indicators in the investigated groups.

Parameter	RUN + WS Group (*n* = 10) M (Min–Max) ± SD	WS Group (*n* = 10) M (Min–Max) ± SD	Control Group (*n* = 10) M (Min–Max) ± SD	*p* (ANOVA on Ranks)	Post Hoc Analysis		
					RUN + WS /WS	WS /CON	RUN + WS /CON
Na^+^ [mmol/L]	141.00 (139–142) ± 0.54	139.50 (139–140) ± 0.45	138.00 (137–139) ± 0.37	0.009 *	0.13	0.06	0.005 *
K^+^ [mmol/L]	4.71 (4.49–4.96) ± 0.12	4.37 (4.15–4.49) ± 0.07	4.25 (4.02–4.6) ± 0.11	0.09	n.s.		
Cl^−^ [mmol/L]	101.05 (100.6–102.4) ± 0.46	101.45 (99–102.8) ± 0.68	99.50 (98.4–101.1) ± 0.41	0.059 *	0.94	0.07	0.02 *
Urea [mmol/L]	5.51 (4.21–6.43) ± 0.45	4.60 (3.77–5.21) ± 0.38	4.34 (4.13–5.22) ± 0.26	0.44	n.s.		
Creatinine [µmol/L]	78.30 (73.8–84.8) ± 3.09	90.75 (77.7–96) ± 0.33	85.40 (77.7–97.4) ± 4.31	0.3	n.s.		
eGFR [ml/min/1.73 m^2^]	90.00 (90–90) ± 1.39	82.00 (78–90) ± 2.15	90.00 (86–90) ± 1.78	0.05	0.02 *	0.11	0.32
Uric acid [µmol/L]	318.80 (273.1–344.5) ± 12.79	398.20 (332.1–422.8) ± 20.76	333.20 (275.5–370.7) ± 18.83	0.04 *	0.01 *	0.14	0.38
Total bilirubin [µmol/L]	11.60 (9.6–15) ± 2.49	7.20 (5.1–10.4) ± 2.15	9.90 (6.2–12.7) ± 3.49	0.13	n.s.		
ALP [U/L]	70.60 (63.5–90.1) ± 4.05	59.60 (67.3–70.7) ± 6.24	99.30 (73.1–110.7) ± 7.2	0.01	0.05	0.02 *	0.04 *
AST [U/L]	26.50 (20.7–29.6) ± 1.73	23.05 (21.4–28) ± 1.57	22.15 (18.4–25.7) ± 3.5	0.36	n.s.		
ALT [U/L]	23.50 (19.9–28) ± 2.02	29.00 (22.5–49.3) ± 5.36	25.75 (17.2–32.4) ± 8.66	0.3	n.s.		
GGT [U/L]	24.00 (18–40) ± 4.41	27.50 (18–45) ± 5.7	24.50 (17–29) ± 3.18	0.68	n.s.		
LDH [U/L]	184.35 (147.5–220) ± 14.94	179.5 (163–185.1) ± 10.01	155.05 (143.6–209.7) ± 12.02	0.39	n.s.		
CK [U/L]	188.15 (129.7–303.1) ± 31.64	146 (100.4–216.7) ± 57.18	115.35 (106–268.3) ± 26.14	0.41	n.s.		
Total protein [g/L]	72.2 (67.6–72.8) ± 1.33	72 (68.2–72.7) ± 1.03	70.25 (68.1–72.2) ± 0.75	0.57	n.s.		
Albumin [g/L]	46.00 (44.4–47.3) ± 0.51	45.05 (43.7–46.9) ± 0.64	44.85 (44–46.2) ± 0.47	0.61	n.s.		
Alpha-1-globulin [g/L]	2.30 (2.2–2.7) ± 0.08	2.40 (2.3–2.8) ± 0.1	2.70 (2.6–2.7) ± 0.04	0.17	n.s.		
Alpha-2-globulin [g/L]	5.20 (4.9–6) ± 0.29	5.00 (4.6–5.3) ± 0.26	5.75 (5.2–6.4) ± 0.2	0.09	n.s.		
Beta-1-globulin [g/L]	4.15 (2.5–4.3) ± 0.27	4.55 (4.1–4.7) ± 0.16	3.95 (3.6–4.2) ± 0.11	0.05	0.12	0.02 *	0.59
Beta-2-globulin [g/L]	3.30 (2.9–4.4) ± 0.32	3.70 (3.3–4.1) ± 0.18	3.25 (2.9–3.6) ± 0.18	0.18	n.s.		
Gamma-globulin [g/L]	9.90 (8.8–11.2) ± 0.66	11.15 (9.4–12.2) ± 6.84	9.10 (8.2–10.8) ± 0.59	0.14	n.s.		
A/G	1.925 (1.58–2.03) ± 0.12	1.715 (1.68–1.78) ± 0.04	1.815 (1.75–2.02) ± 0.06	0.32	n.s.		
IgA [g/L]	2.40 (1.6–2.7) ± 0.33	2.40 (2.2–3) ± 0.21	1.90 (1.4–2.2) ± 0.24	0.12	n.s.		
IgG [g/L]	11.15 (10–12.2) ± 0.65	11.90 (10.5–12.2) ± 0.49	11.25 (9.2–13.9) ± 0.77	0.93	n.s.		
IgM [g/L]	0.80 (0.6–1) ± 0.13	0.95 (0.5–1.5) ± 0.16	0.80 (0.7–1) ± 0.11	0.94	n.s.		
CRP [mg/L]	0.46 (0.23–0.64) ± 0.3	0.93 (0.48–1.08) ± 0.26	0.39 (0.28–0.67) ± 0.16	0.26	n.s.		

M—median; RUN—running; WS—winter swimming; CON—control; eGFR—estimated glomerular filtration rate; ALP—alkaline phosphatase; AST—aspartate transaminase; ALT—alanine transaminase; GGT—gamma-glutamyltransferase; LDH—lactate dehydrogenase; CK—creatine kinase; A/G—albumin/globulin ratio; CRP—C-reactive protein; n.s.—no significant difference. * Significant difference (*p* < 0.05).

## Data Availability

The datasets used and/or analyzed during the current study are available from the corresponding author on reasonable request.
